# Augmenting Weak Semantic Cognitive Maps with an “Abstractness” Dimension

**DOI:** 10.1155/2013/308176

**Published:** 2013-06-12

**Authors:** Alexei V. Samsonovich, Giorgio A. Ascoli

**Affiliations:** Krasnow Institute for Advanced Study, George Mason University, 4400 University Drive MS 2A1, Fairfax, VA 22030-4444, USA

## Abstract

The emergent consensus on dimensional models of sentiment, appraisal, emotions, and values is on the semantics of the principal dimensions, typically interpreted as valence, arousal, and dominance. The notion of weak semantic maps was introduced recently as distribution of representations in abstract spaces that are not derived from human judgments, psychometrics, or any other a priori information about their semantics. Instead, they are defined entirely by binary semantic relations among representations, such as synonymy and antonymy. An interesting question concerns the ability of the antonymy-based semantic maps to capture all “universal” semantic dimensions. The present work shows that those narrow weak semantic maps are not complete in this sense and can be augmented with other semantic relations. Specifically, including hyponym-hypernym relations yields a new semantic dimension of the map labeled here “abstractness” (or ontological generality) that is not reducible to any dimensions represented by antonym pairs or to traditional affective space dimensions. It is expected that including other semantic relations (e.g., meronymy/holonymy) will also result in the addition of new semantic dimensions to the map. These findings have broad implications for automated quantitative evaluation of the meaning of text and may shed light on the nature of human subjective experience.

## 1. Introduction

The idea of representing semantics geometrically is increasingly popular. Many mainstream approaches use vector space models, in which concepts, words, documents, and so forth are associated with vectors in an abstract multidimensional vector space. Other approaches use manifolds of more complex topology and geometry. In either case, the resultant space or manifold togetheD:\Finalization\VLSI\861691\861691r with its allocated representations is called a *semantic space* or a *semantic (cognitive) map*. Examples include spaces constructed with Latent Semantic Analysis (LSA) [1] and Latent Dirichlet Allocation (LDA) [2], as well as many related techniques, for example, ConceptNet [3, 4]. Other examples of techniques include Multi-Dimensional Scaling (MDS) [5], including Isomap [6], and related manifold-learning techniques [7], Gardenfors' conceptual spaces [8], very popular in the past models of self-organizing feature maps, and more.

The majority of these approaches are based on the idea of a dissimilarity metrics, which is to capture semantic dissimilarity between representations (words, documents, concepts, etc.) with a geometrical distance between associated space elements (points or vectors). In other words, the metrics that determines the allocation of representations in space is a function of their semantic dissimilarity. In this case, two representations allocated at close points in space must have similar semantics and vice versa: two representations with similar semantics must be close to each other in space. Conversely, representations unrelated to each other must be separated by significant distance.

We introduced the term “weak semantic cognitive mapping” to denote an alternative approach, exploited here, which is not based on dissimilarity [9–11]. The idea is not to separate all different meanings from each other (like in MDS), nor to allocate them based on their individual semantic characteristics given a priori (as in LSA), but rather to arrange them in space based on their mutual semantic relations. The notion of weak semantic cognitive maps was originally introduced in a narrow sense, where these relations were limited to synonymy and antonymy only [9–11]. In a more general sense, as discussed below, weak semantic cognitive maps may capture other binary semantic relations as well, including hypernymy-hyponymy, holonymy-meronymy, troponymy, causality, and dependence. 

While the understanding of dissimilarity as the basis of antonymy is widespread, many examples of the dictionary antonym pairs used in our analysis suggest that dissimilarity and antonymy are distinct notions. Most unrelated words may be considered dissimilar (e.g., “apple” and “inequality”), yet do not constitute antonym pairs. In contrast, antonym pairs include words that are related to each other and in a certain sense are similar to each other in their meaning and usage, for example, king and queen, major and minor, and ascent and descent. It appears that most antonym pairs (at least in the dictionaries that we used) are consistent with the notion of “opposite” rather than “dissimilar.”

More generally, the method of weak semantic mapping is essentially different from most vector-space-based approaches including LSA, LDA, MDS, and ConceptNet [1–4], primarily because there is no a priori attribution of semantic features to representations in the constructive definition of the map. Only relations, but not semantic features, are given as input. As a result, semantic dimensions of the map that are not predefined to emerge naturally, starting from a randomly generated initial distribution of words in an abstract space with no a priori given semantics and following the strategy to pull synonyms together and antonyms apart [10, 11] (see Section 2: Methods). In contrast to LSA, principal component analysis is used here to reveal the main emergent semantic dimensions at the final stage only. The advantage of the antonymy-based weak semantic cognitive map compared to “strong” maps based on dissimilarity metrics is that its dimensions have clearly identifiable semantics (naturally given by the corresponding pairs of antonyms) that are domain-independent. For example, the notion of “good versus bad” that corresponds to the first principal component applies to all domains of human knowledge. 

Interestingly, semantics of the emergent dimensions of antonym-based weak semantic cognitive maps are closely related to those of another broad category of “dimensional models” of affects [12] that attempt to capture human emotions, feelings, affects, appraisals, sentiments, and attitudes. Examples range from original classical models such as Osgood's semantic differential [13], Russell's circumplex [14], and Plutchik's wheel [15] to many more recent derivative integrated frameworks, like PAD (pleasure, arousal, and dominance) [16], ANEW (Affective Norms for English Words) [17], EPA (evaluation, potency, and arousal) [18], and a recent 3D model linking emotions to main neurotransmitters [19]. These dimensional models are usually derived from human experimental studies involving psychometrics or introspective judgment evaluated on the Likert scale [20]. While these models provide the most common bases for opinion mining or sentiment analysis [21], the weak semantic map is more complete in the sense that (i) it assigns values to all words, not only to emotionally meaningful words, (ii) it measures semantics associated with all antonym pairs, not only emotionally meaningful antonym pairs, and therefore is applicable to all domains of knowledge, and (iii) its dimensions are orthogonal and independent of each other. The combination of these features makes weak semantic maps extremely valuable for numerous applications.

It is surprising that the well-known dimensions of the semantic differential, PAD, EPA, and related models can be recognized in the main principal components (PC) of the above cited weak semantic map, where PC1 is related to valence, PC2 to arousal, and PC3 to dominance [11]. (This correspondence is approximate, because the principal components have zero correlations with each other, while the variables of, e.g., ANEW are strongly correlated.) For example, “love” and “joy” have top values of valence in the affective database ANEW and also top values of PC1 of weak semantic cognitive map. Words like “anger” and “excitement” have top values of arousal in the affective database ANEW and also top values of PC2 in weak semantic cognitive map. This correspondence is consistent in weak semantic maps constructed based on different corpora in several major languages [11]. The observation is unexpected, because the weak semantic map is not derived from any semantic features of words given a priori, and is not explicitly related to emotions and feelings by its construction. In fact, any pair of antonyms defines a map dimension, including antonym pairs that are not associated with affects, for example, “abstract-specific.” It is also surprising that the weak semantic map is low-dimensional: the number of PCs that account for 95% of the variance of the multidimensional distribution typically varies from 4 to 6, depending on the corpus [11].

How complete is the weak semantic map narrowly defined only by antonym pairs? Certainly at least some semantic differences cannot be captured by antonymy relations, because not all concepts have antonyms (e.g., the number 921714083). Here we address a different question: whether all universal semantic dimensions can be captured by antonymy relations. For example, it may seem obvious that causality cannot be captured by antonymy. However, the issue is nontrivial, as there are many examples of causally related antonyms (e.g., attack-defend, begin-end, send-receive, and even cause-effect). Thus, two logical possibilities stand.

1. Antonym-based semantic maps separate representations along all semantic dimensions that make sense for all domains of knowledge. Thus, if there is a semantic characteristic *X* that makes sense for all domains of knowledge such that some concepts can be characterized as having more *X* than others, then there is a direction on the narrow weak semantic map along which those concepts are separated based on their value of *X*.
2. The alternative: there is at least one general semantic characteristic *X* defined for all domains that is ignored by the antonym-based weak semantic map. In other words, the variance in *X* measured across all concepts is not accounted by the map coordinates of concepts, and vice versa, no significant part of the variance of the map can be accounted by *X*.

Here we argue for (2), quantifying the notion of “abstractness” (or ontological generality) as an example of *X*. Our technical definition of “abstractness” is based on hyponym-hypernym relations among words. 

**Figure 1:**
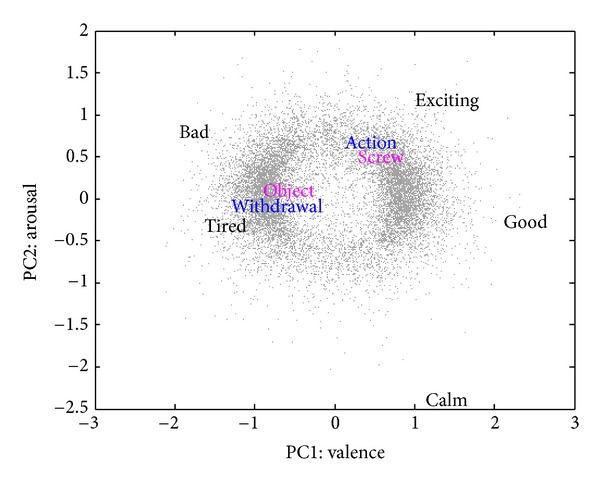
A sample from the antonymy-based weak semantic cognitive map constructed by Samsonovich and Ascoli [11]. Grey dots show all 15,783 words from the MS Word English dictionary. Similar results were obtained by WordNet. Words shown in color are examples of hypernym-hyponym pairs: “action-withdrawal” and “object-screw.” Selected examples illustrate that there is no clear separation of hypernyms and hyponyms on the map.

Before presenting results of the computational study, we briefly discuss the hypothesis at an intuitive level. While “abstract-specific” is a pair of antonyms, which corresponds to a direction on the narrow weak semantic map, the two antonyms “abstract” and “specific” themselves have approximately the same measure of “abstractness” (the *X* value) associated with them. Intuitively, this observation must hold for most antonym pairs, because antonyms pairs do not typically constitute a hypernym-hyponym couple. Therefore, it is unlikely that there is a hyperplane on the map that separates more abstract from more specific words. Therefore, we do not expect to find a dimension of the map based on synonyms and antonyms that could separate words by “abstractness” (see Figure 1). In contrast, there is a hyperplane (PC1 = 0) that separates “good” and “bad” words and a hyperplane (PC2 = 0) that separates “calming” and “exciting” words. That is to say, “good words” tend to be synonyms of the word “good,” but “abstract words” are not synonyms of the word “abstract” or of each other.

## 2. Methods

### 2.1. Weak Semantic Cognitive Mapping

The general idea of semantic cognitive mapping is to allocate representations (e.g., words) in an abstract space based on their semantics. This paradigm is common for a large number of techniques overviewed in Introduction. While most studies in semantic cognitive mapping are based on the notion of a dissimilarity metrics and/or on a set of semantic features given a priori, weak semantic mapping ignores dissimilarity as well as any individually predefined semantics.

The algorithm for antonymy-based weak semantic mapping is described in our previous work [11]. The semantic space is created by minimization of the “energy” of the entire distribution of words on the map, starting from a random distribution. Then, the emergent semantics of the map dimensions are defined by the entire distribution of representations on the map and typically are best characterized by the pairs of antonyms that are separated by the greatest distance along the given dimension. The main semantic dimensions are defined by the principal components of the emergent distribution of words on the map. Semantics associated with the first three PCs can be characterized as “good” versus “bad” (PC1), “calming, easy” versus “exciting, hard” (PC2), and “free, open” versus “dominated, closed” (PC3) [11]. When limited to affects, these semantics approximately correspond to the three PAD dimensions: pleasure, arousal, and dominance.

More precisely, the narrow weak semantic cognitive map is a distribution of words in an abstract vector space (with no semantics preassociated with its elements or dimensions) that minimizes the following energy function [11]:

(1) $$\begin{array}{c} H\left(x\right)=-\frac{1}{2}\sum_{i,j\;=1}^{N}W_{ij}x_{i}\cdot x_{j}+\frac{1}{4}\sum_{i\;=1}^{N}{\left|x_{i}\right|}^{4},\;x\in {ℜ}^{N}⊗{ℜ}^{D}. \end{array}$$

Here **x**_*i*_ is a *D*-vector representing the *i*th word (out of *N*). The *W*_*ij*_ entries of the symmetric relation matrix equal +1 for pairs of synonyms, –1 for pairs of antonyms, and zero otherwise. *D* is set to any integer (e.g., 100) that is substantially greater than the number of resulting significant principal components of the distribution, which typically ranges from 4 to 6 and determines the dimensionality of the map. In this case the choice of *D* does not change the outcome. The energy function (1) follows the principle of parsimony: it is the simplest analytical expression that creates balanced forces of desired signs between synonyms and antonyms, preserves symmetries of semantic relations, and increases indefinitely at the infinity, keeping the resultant distribution localized near the origin of coordinates.

The procedure is that the initial coordinates of all words are sampled by a random number generator. Then the energy (1) minimization process starts that pulls synonym vectors together and antonym vectors apart. Then principal component analysis is used to reveal the main emergent semantic dimensions of the optimized map [10, 11]. Thus, the initial space coordinates are not associated with any semantics a priori: instead, words are allocated randomly in an abstract multidimensional space. In contrast, the starting point of traditional techniques based on LSA [1, 22] is a feature space, where dimensions have definite semantics a priori. 

The representative weak semantic map shown in Figure 1 includes *N* = 15, 783 words and was constructed based on the dictionary of English synonyms and antonyms available as part of Microsoft Word (MS Word) [11]. A similar map was also constructed using WordNet in the same work [11] and is also used in this study, together with maps constructed in [11] for other languages. Figure 1 represents the first two PCs of the distribution of words on the map constructed using the English MS Word thesaurus. The axes of the map are defined by the PCs. Selected words shown on the map in black at their map locations characterize the semantics of the map. The two hypernym-hyponym pairs, “object-screw” (shown in pink) and “action-withdrawal” (in blue), illustrate the map inability to capture the “abstractness” dimension, confirmed quantitatively by correlation analysis in the next section. It should be pointed out here that the negative valence of “object” can be attributed to the meaning of the verb “object” that is merged with the noun “object” on this string-based semantic map.

### 2.2. Measuring the “Abstractness” of Words

Here we refer to the “abstractness” of a concept as its ontological generality. The WordNet database contains information that allows us to arrange English words on a line according to their “abstractness” (or ontological generality). This information is contained in the hyponym-hypernym relations among words. The goal is to separate hypernym-hyponym pairs in one dimension tentatively labeled “abstractness,” so that each hyponym has a lower “abstractness” value compared to its hypernyms. Given a consistent hierarchy, a solution would be, for example, to interpret the order of a word in the hierarchy as a measure of its “abstractness.” Unfortunately, the system of hyponym-hypernym relations among words available in WordNet is internally inconsistent: it has numerous loops and conflicting links. Therefore, we use an optimization approach analogous to the antonymy-based weak semantic mapping based on (1). The underlying idea is to give each word *i* its “abstractness” coordinate *x*_*i*_ in such a way that the overall correlation between the difference in word “abstractness” coordinates *x* and the reciprocal hypernym-hyponym relations of the two words is maximized. Unfortunately, an energy function similar to *H* (1) cannot be used here, because the symmetry of hypernym-hyponym relations is different from the symmetry of antonym and synonym relations. Nevertheless, we showed in previous work [23] that the goal can be achieved by using the following definition of word “abstractness” values {*x*}:

(2) $$\begin{array}{c} \vec{x}=\underset{{\mathbb{R}}^{n}}{\text{argmin}}\left[\sum_{i,j\;=1}^{n}{W_{ij}\left(x_{i}-x_{j}-1\right)}^{2}+\mu \sum_{i\;=1}^{n}x_{i}^{2}\right], \end{array}$$

where *n* is the number of words, *μ* is a regularization parameter, and *W*_*ij*_ = 1 if the word *i* is a hypernym of the word *j* and zero otherwise. Here the first sum is taken over all ordered hyponym-hypernym pairs. 

The publicly available WordNet 3.0 database (http://wordnet.princeton.edu/) was used in this study. The hypernym-hyponym relations among *n* = 124, 408 English words were extracted from the database as a connected graph defining the matrix *W*, which was used to compute the energy function (2). Optimization was carried out with standard MATLAB functions, as described in [23].

## 3. Results

### 3.1. Measuring Correlations of Augmented Map Dimensions

**Figure 2:**
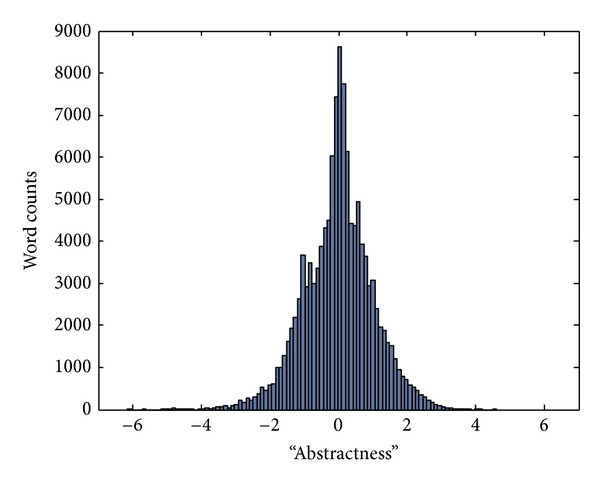
Distribution histogram of the 124,408 WordNet 3.0 words along the “abstractness” dimension.

The one-dimensional semantic map of “abstractness” was computed as described in Section 2. The resultant distribution of 124,408 WordNet words in one dimension is shown in Figure 2. The two ends of the sorted list of words along their “abstractness” are given in Table 1.

This map was then combined with several antonymy-based weak semantic maps that are previously constructed [11]. The “abstractness” map was merged with any given narrow weak semantic map as the following. First, the set of words was limited to those that are common for both maps. Then, the augmented map was defined as a direct sum of the two vector spaces; that is, the “abstractness” dimension was added as a new word coordinate.

**Figure 3:**
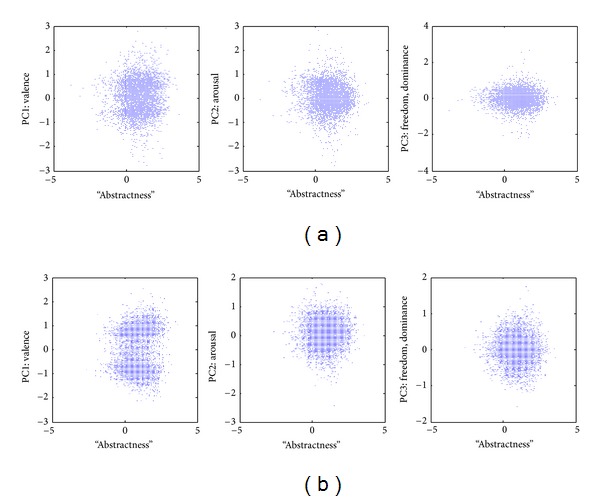
Correlations of “abstractness” with principal components of the antonymy-based weak semantic cognitive maps. (a) The map constructed using WordNet 3.0; (b) the map constructed using the Microsoft Word thesaurus.

The resultant augmented maps were used to compute the correlation between “abstractness” and other map dimensions. The main question was how, if at all is the new “abstractness” dimension related to the principal components of the antonymy-based weak semantic map? Figure 3 illustrates the scatterplots of word “abstractness” values derived from WordNet with the dimensions of narrow weak semantic maps derived from WordNet data (Figure 3(a)) and from MS Word (Figure 3(b)). The Pearson correlation coefficient *R* and the corresponding accounted variance *R* ^2^ are given in Table 2 for each PC.

**Figure 4:**
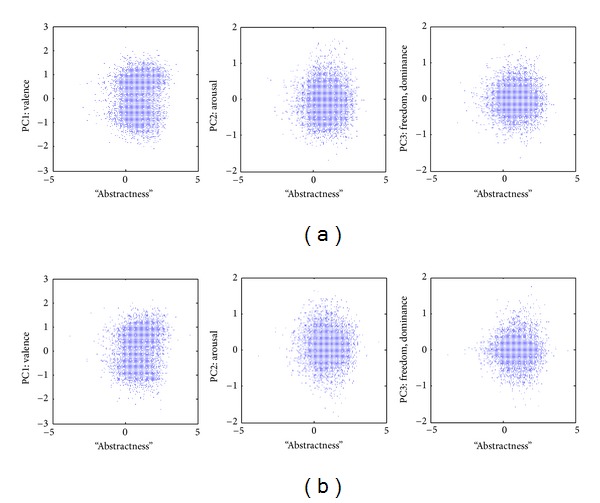
Correlations of “abstractness” with principal components of the antonymy-based weak semantic cognitive maps in other languages. (a) The map constructed using the French dictionary of MS Word. (b) The map constructed using the German dictionary of MS Word.

Similar results were obtained for augmented weak semantic maps in other languages (constructed based on the MS Word thesaurus as described in [11]): French (Figure 4(a)) and German (Figure 4(b)). Automated Google translation was used to merge maps in different languages.

In all cases “abstractness” is only positively correlated with valence (*P* < 10^−8^ in all corpora), while none of the correlation coefficients with the other two dimensions (arousal and freedom) are statistically significant in a consistent way across corpora. Even in the case of valence, the correlation coefficient remains small (Table 2). This finding is further addressed in Section 4.

Overall, the results (Figures 3 and 4) show that the new “abstractness” dimension is practically orthogonal to the narrowly defined weak semantic map dimensions. Indeed, in most cases the correlation is not significant. In the minority of the cases where the correlation is statistically significant, the correlation coefficient is sufficiently small as to become marginal. Specifically, little information is lost by disregarding the fraction of the variance of the distribution of words on the weak semantic map accounted by the word “abstractness” or, vice versa, the fraction of the variance in the word “abstractness” accounted by the weak semantic map dimensions (Table 2).

In conclusion, the previous weak semantic map dimensions do not account for a substantial fraction of variance in “abstractness,” and word “abstractness” values do not account for a substantial fraction of variance in the distribution of words on antonymy-based weak semantic maps.

### 3.2. Examples of Document Mapping with the Augmented Semantic Map

Traditionally, only the valence dimension is used in sentiment analysis. At the same time, other dimensions including “abstractness” are frequently indicated as useful (e.g., [24]). We previously applied the weak semantic map to analysis of Medline abstracts [25]. As an extension of that study, we now applied the augmented semantic map to analyze various kinds of documents. 

Using the MS Word English narrow weak semantic map merged with the WordNet-based “abstractness” map, this part of the study asked the following key research questions: how informative is the new dimension compared to familiar dimensions at the document level? Specifically, how well are different kinds of documents separated from each other on the augmented map compared to the narrow weak semantic map? How capable is the new “abstractness” dimension compared to antonymy-based dimensions in terms of document separation? Being aware of more advanced methods of sentiment analysis [21, 26], here we adopted the simplest “bag of words” method (computing the “center of mass” of words in the document, not to be confused with LSA). This parsimonious choice is justified because at this point we are interested in assessing the value of the new dimension compared to familiar dimensions of the narrow weak semantic map, rather than achieving practically significant results.

**Figure 5:**
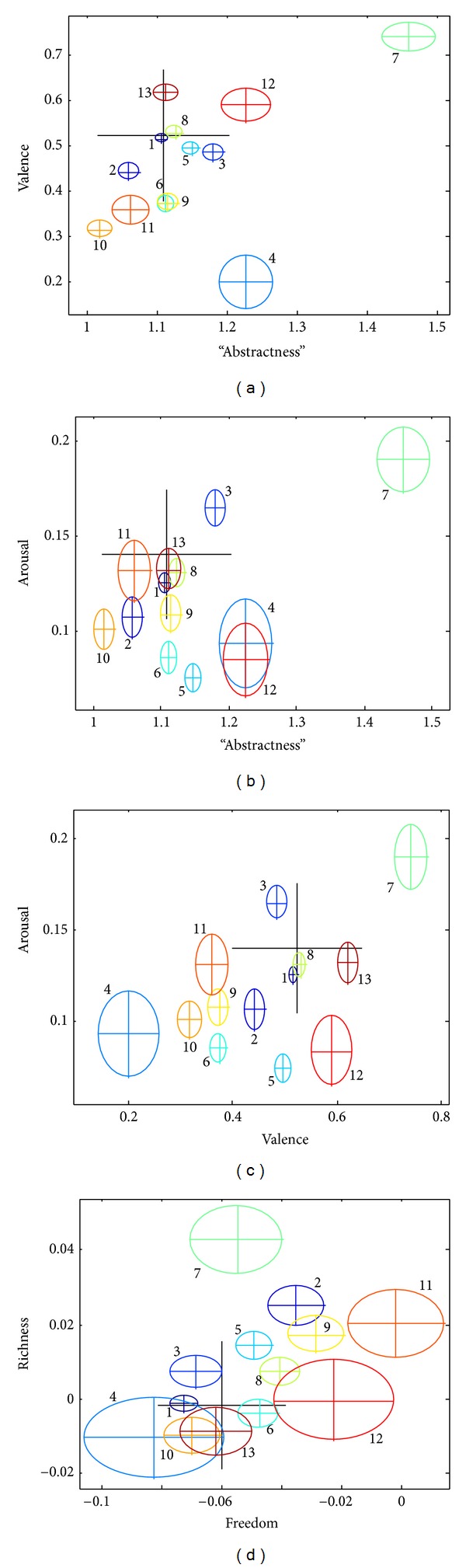
Representations of 13 documents (details in the text) on the augmented semantic map. The Pearson correlation coefficient *R* and the corresponding *P* value were computed for each panel. None of the correlations are significant. (a) Valence versus “abstractness,” *R* = 0.54, *P* = 0.06. (b) Arousal versus “abstractness,” *R* = 0.50, *P* = 0.09. (c) Arousal versus valence, *R* = 0.54, *P* = 0.057. (d) Richness (PC4) versus freedom (PC3), *R* = 0.46, *P* = 0.12.

For each document, the average augmented map coordinates of all words were computed, together with the standard error in each dimension. The results are represented in Figure 5 by crossed ovals, with the center of the cross representing the average and the size of the oval representing the standard error (i.e., the standard deviation divided by the square root of the number of identified words). The large black crosses in each panel represent the average of all words in the dictionary weighted by their overall usage frequency, not limited to materials of this study and derived as in [11]. Colors and numbers of ovals in Figure 5 correspond to RGB values and item numbers given in the following list of corpora:

1. Project Gutenberg's A Text-Book of Astronomy, by George C. Comstock (http://www.gutenberg.org/files/34834/34834-0.txt), 9626 words, rgb = (0, 0, 6);
2. Martha Stewart Living Radio Thanksgivings Hotline Recipes 2011 (http://www.hunt4freebies.com/free-martha-stewart-thanksgiving-recipes-ebook-download), 2091 words, rgb = (0, 0, 9);
3. Al Qaida Inspire Magazine Issue 9 (http://www.en.wikipedia.org/wiki/Inspire_(magazine)), 2555 words, rgb = (0, 2, 10);
4. A suicide blog (http://www.tumblr.com/tagged/suicide-blog), 387 words, rgb = (0, 5, 10);
5. 152 Shakespeare sonnets [27], 4170 words, rgb = (0, 8, 10);
6. The Hitchhiker's Guide to the Galaxy, by Douglas Adams (http://www.paulyhart.blogspot.com/2011/10/hitchhikers-guide-to-galaxy-text_28.html), 4187 words, rgb = (1, 10, 9);
7. 10 abstracts of award-winning NSF grant proposals (downloaded from http://www.nsf.gov/awardsearch), 585 words, rgb = (4, 10, 6);
8. 196 reviews of the film “Iron Man”, 2008 (http://www.mrqe.com/movie_reviews/iron-man-m100052975/), 3902 words, rgb = (8, 10, 2);
9. 170 reviews of the film “Superhero Movie”, 2008 (http://www.mrqe.com/movie_reviews/superhero-movie-m100071304/), 2204 words, rgb = (10, 9, 0);
10. 160 reviews of the film “Prom Night”, 2008 (http://www.mrqe.com/movie_reviews/prom-night-m100076394/), 2114 words, rgb = (10, 6, 0);
11. 47 anecdotes of/about famous scientists (retrieved from http://jcdverha.home. xs4all.nl/scijokes/10.html), 919 words, rgb = (10, 3, 0);
12. transcript of Obama's speech at the DNC on September 6, 2012 (foxnews.com/politics/2012/09/06/transcript-obama-speech-at-dnc), 491 words, rgb = (10, 0, 0);
13. “Topological strings and their physical applications,” by Andrew Neitzke and Cumrun Vafa (http://www.arxiv.org/abs/hep-th/0410178v2), 1909 words, rgb = (7, 0, 0).

The selected documents are mostly well separated in 3 dimensions, including valence (PC1), arousal (PC2), and “abstractness” (Figure 5). At the same time, the ovals more frequently overlap on the plane freedom-richness (PC3-PC4). Visually, “abstractness” is approximately as efficient as valence (PC1) in its ability to separate documents and appears to be more efficient than other dimensions; however, the oval separation on the valence-arousal projection (Figure 5(c)) looks slightly better than on the valence-“abstractness” projection (Figure 5(a)). This observation suggests that disregarding “abstractness” may not significantly affect the quality of results, while disregarding valence would substantially impair the quality of document separation (e.g., on the “abstractness-”arousal plane, Obama's speech overlaps substantially with the suicide blog, while valence separates the two documents significantly). 

Differences between the above 13 documents along these 5 dimensions were quantified with analysis of variance. Specifically, the MANOVA *P* value was 0.027, suggesting that all five semantic dimensions are mutually independent in characterizing the selected 13 corpora. Moreover, in order to compare how informative different semantic dimensions are relative to each other, two sets of characteristics were computed (Table 3), namely, (i) the ANOVA *P* values to reject the null hypothesis that all 13 corpora have the same mean in each selected semantic dimension and (ii) the MANOVA *P* values to reject the null hypothesis that the means of all 13 corpora belong to a low-dimensional hyperplane within the space of all but one semantic dimensions.

These results can be interpreted as follows. The lower the *P* value for ANOVA is, the more informative the selected semantic dimension is. On the contrary, the lower the *P* value for MANOVA is, the less informative the selected semantic dimension is, because MANOVA was computed in the space of all semantic dimensions except the one selected. Therefore, results represented in Table 3 indicate that “abstractness” (dimension 0) is nearly as informative as valence (dimension 1) and could be more informative than arousal (dimension 2, based on ANOVA only), freedom (dimension 3), and richness (dimension 4). More data are needed to verify this interpretation.

## 4. Discussion

Statistical analysis indicates that “abstractness” is positively (if marginally) correlated with valence consistently across corpora, which is not the case with other semantic dimensions. On the one hand, the amount of variance in the distributions of words that can be attributed to interaction between valence and “abstractness” is not substantial (only 2% of variance or less); therefore, the two dimensions can be considered orthogonal for practical purposes. On the other hand, the consistent significance of this negligibly small correlation across datasets and languages indicates that there may be a universal factor responsible for it. This factor could be the usage frequency of words that affects the probability of word selection for dictionaries. Stated simply, abstract positive words and specific negative words are used more frequently than abstract negative words and specific positive words. Specifically, our previous study [11] showed that the mean valence (normalized to unitary standard deviation) of all words weighted by their usage frequency is significantly positive (0.50 using frequency data from a database of Australian newspapers and 0.59 using frequency data from the British National Corpus). Using the results in the present study, the mean normalized “abstractness” is between 0.99 (weighted by “Australian” frequency) and 1.39 (weighted by “British” frequency). An equivalent explanation is that abstract words and positive words are both used more frequently than specific words and negative words. Specifically, the correlation with frequency is small but significantly positive both for valence (0.064 Australian, 0.061 British) and for “abstractness” (0.036 Australian, 0.019 British). This interpretation is consistent with data at the level of documents (Figure 5(a)), where the correlation coefficient is even higher, yet not significant (not shown). Another potential source of correlation is the selection of words for inclusion in dictionaries. It seems, however, counterintuitive that the overall picture should be affected by marginal inclusions of rare words. Nevertheless, it would be interesting to check elsewhere how the correlation changes across sets of words found in various types of documents.

The method of weak semantic mapping is an alternative to other vector-space-based approaches including LSA, LDA, MDS, and ConceptNet [1–4], primarily because (i) no semantic features of words are given as input and (ii) the abstract space of the map has no semantics associated a priori with its dimensions. It is therefore not surprising that emergent semantic features (dimensions) in weak semantic mapping are substantially different from emergent semantic dimensions obtained by LSA and related techniques: the latter are typically domain specific and harder to interpret [22].

From another perspective, it is interesting that emergent semantic dimensions of a weak semantic map are so familiar. All generally accepted dimensional models of sentiment, appraisal, emotions and values, attitudes, feelings, and so forth converge on semantics of their principal dimensions, typically interpreted as valence, arousal, and dominance [12–14, 16–18]. Antonymy-based weak semantic mapping appears to be consistent with this emergent consensus [9–11], despite the stark difference in methodologies (human judgment or psychometrics versus automated calculations based on subject-independent data). The number of semantic dimensions, or factors, used in the literature varies from 2 to 7, which roughly corresponds to the variability in the number of significant principal components of the narrow weak semantic map [11]. Why do antonyms relating to the “dimensional models" of affect, and not others, make for good PCs? This interesting question remains open and should be addressed by future studies.

The present study unambiguously demonstrates the inability of narrow weak semantic maps to capture all universal semantic dimensions. Here we presented one dimension, “abstractness,” that is not captured by “antonymy-” defined weak semantic maps. This is due to the fact that, in general, hypernym-hyponym pairs are not antonym pairs and vice versa. Therefore, hypernym-hyponym relations cannot be captured with the map defined by antonym relations, and the map needs to be augmented. The example of “abstractness” that we found is probably not unique: we expect a similar outcome for the holonym-meronym relation, which will be addressed elsewhere. Our previous results indicated that antonym relations are essential for weak semantic mapping, while synonym relations are not [28].

Thus, the present work shows that narrow weak semantic maps (and related dimensional models of emotions) are not complete in this sense and need to be augmented by including other kinds of semantic relations in their definition. A question remains open as to whether any augmented semantic map may be considered complete—or there will always be new semantic dimensions that can be added to the map. We speculate that there exists a complete finite-dimensional weak semantic map. Moreover, the number of its dimensions can be relatively small. This is because the number of distinct semantic relationships in natural language is limited, as is the number of primary categories [29], or the number of primary semantic elements of metalanguage known as semantic primes [31, 32]. This notion of “completeness,” however, may only be applicable to a limited scope, for example, all existing natural languages.

We found that hyponym-hypernym relations induce a new semantic dimension on the weak semantic map that is not reducible to any dimensions represented by antonym pairs or to the traditional PAD or EPA dimensions. Its tentative labeling as “abstractness” or ontological generality, however, remains speculative. In any case, it is not our ambition here to define the notion of “abstractness” or to establish a precise connection between the real notion of abstractness and our new “abstractness” dimension, a topic that should be addressed elsewhere.

Findings of this study have broad implications for automated quantitative evaluation of the meaning of text, including semantic search, opinion mining, sentiment analysis, and mood sensing, as exemplified in Figure 5 and Table 3. While multidimensional approaches in opinion mining are nowadays popular, the problem is finding good multidimensional ranking of all words in the dictionary. Traditional bootstrapping methods (e.g., based on cooccurrence of words) to extend the ranking of positivity from a small subset of words to all words may not work, for example, for “abstractness.” The approach presented here should be useful for such applications. 

Finally, we speculate that this approach may shed light on the nature of human subjective experience [30] by revealing fundamental semantics of qualia as PCs of the weak semantic cognitive map. In addition, we suggest other connections of our findings, for example, to semantic primes [31, 32].

## Figures and Tables

**Table 1:** The tails of the list of 124,408 words sorted by “abstractness” in descending order.

The beginning of the list	The end of the list
Entity	Chain wrench
Physical entity	Francis turbine
Psychological feature	Tricolor television tube
Auditory communication	Tricolor tube
Unmake	Tricolour television tube
Cognition	Tricolour tube
Knowledge	Edmontonia
Noesis	*Coelophysis *
Natural phenomenon	*Deinocheirus *
Ability	*Struthiomimus *
Social event	*Deinonychus *
Craniate	Dromaeosaur
Vertebrate	*Mononychus olecranus *
Higher cognitive process	*Oviraptorid *
Physiological property	*Superslasher *
Mammal	*Utahraptor *
Mammalian	*Velociraptor *

**Table 2:** Pearson correlation coefficient *R* and the corresponding accounted variance (*R* ^2^) of “abstractness” with PC1: valence, PC2: arousal, and PC3: freedom/dominance, measured in four augmented maps constructed based on WordNet 3.0 and the MS Word English, French, and German thesauri.

	PC1: valence		PC2: arousal		PC3: freedom, dominance	
	*R*	*R* ^2^	*R*	*R* ^2^	*R*	*R* ^2^
WordNet	0.09	0.8%	−0.07	0.5%	−0.01	0% (NS)
MS Word English	0.12	1.4%	0.01	0% (NS)	−0.03	0.1% (NS)
MS Word French	0.11	1.2%	0.02	0% (NS)	0.01	0% (NS)
MS Word German	0.14	2.0%	−0.02	0% (NS)	0	0% (NS)

**Table 3:** ANOVA and MANOVA *P* values for selected semantic dimensions characterizing the means of the 13 corpora. Dimensions are numbered as follows: 0, “abstractness”, 1, PC1 (valence), 2, PC2 (arousal), 3, PC3 (freedom/dominance), and 4, PC4 (richness).

Semantic Dimension	0	1	2	3	4
One dimension, ANOVA	1.2*e* − 36	5.9*e* − 57	3.1*e* − 15	2.1*e* − 7	6.2*e* − 11
All but one, MANOVA	0.018	0.040	0.041	5.1*e* − 7	1.4*e* − 7
